# Lymphatic and Blood Endothelial Extracellular Vesicles: A Story Yet to Be Written

**DOI:** 10.3390/life12050654

**Published:** 2022-04-28

**Authors:** Johanna Trisko, Johanna Fleck, Silvio Kau, Johannes Oesterreicher, Wolfgang Holnthoner

**Affiliations:** 1 Ludwig-Boltzmann-Institute for Traumatology, The Research Center in Cooperation with AUVA, A-1200 Vienna, Austria; johanna.trisko@trauma.lbg.ac.at (J.T.); johanna.fleck@trauma.lbg.ac.at (J.F.); silvio.kau@trauma.lbg.ac.at (S.K.); johannes.oesterreicher@trauma.lbg.ac.at (J.O.); 2 Austrian Cluster for Tissue Regeneration, A-1200 Vienna, Austria; 3 Institute of Morphology, University of Veterinary Medicine Vienna, A-1210 Vienna, Austria

**Keywords:** vasculature, vascular endothelial cell, exosome, microvesicle, apoptotic body, EV biogenesis, cargo, miRNA, biomarker, vasculopathy

## Abstract

Extracellular vesicles (EVs), such as exosomes, microvesicles, and apoptotic bodies, are cell-derived, lipid bilayer-enclosed particles mediating intercellular communication and are therefore vital for transmitting a plethora of biological signals. The vascular endothelium substantially contributes to the circulating particulate secretome, targeting important signaling pathways that affect blood cells and regulate adaptation and plasticity of endothelial cells in a paracrine manner. Different molecular signatures and functional properties of endothelial cells reflect their heterogeneity among different vascular beds and drive current research to understand varying physiological and pathological effects of blood and lymphatic endothelial EVs. Endothelial EVs have been linked to the development and progression of various vascular diseases, thus having the potential to serve as biomarkers and clinical treatment targets. This review aims to provide a brief overview of the human vasculature, the biology of extracellular vesicles, and the current knowledge of endothelium-derived EVs, including their potential role as biomarkers in disease development.

## 1. The Human Vasculature and Endothelial Cell Heterogeneity

The human vascular system can be broadly separated into the blood and the lymphatic system. The blood vasculature is responsible for the active supply and distribution of blood and its components, whereas the lymphatic system removes accumulating interstitial fluid to ensure tissue homeostasis. Thus, the formation, maintenance, and remodeling of a functioning vascular network is essential for oxygen and nutrient supply as well as lymphatic fluid drainage in the healthy human body [1]. Vasculogenesis describes the formation of new vessels from endothelial progenitor cells [2]. In contrast, angiogenesis refers to the formation of new vessels splitting and sprouting from preexisting ones [3]. Endothelial cells (ECs) arise from the mesodermal stem cell lineage. Mesodermal progenitor cells develop to angioblasts under the influence of a group of transcription factors including E26 transformation-specific (ETS) [4]. Specification into endothelial and hematopoietic lineage is primarily driven through the expression of transcription factors Etv2 and Npas4l [5]. During this process, activation of distinct signaling networks leads to further differentiation into arterial, venous, and lymphatic subtypes [1]. The specification into arterial or venous fate is further driven by regulators such as SoxF transcription factors, Notch receptor proteins, bone morphogenic proteins (BMPs), and transforming growth factor-beta (TGF-ß) [5]. The upregulation of Notch has been shown to lead simultaneously to the expression of arterial markers and the downregulation of venous ones [6]. By suppressing of Notch signaling, the nuclear receptor Coup-TFII acts as a key regulator of venous identity [4]. Regarding the “second” vascular system, lymphatic cell commitment is influenced by vascular endothelial growth factor-C (VEGF-C)/vascular endothelial growth factor receptor-3 (VEGFR3, FLT4)-signaling and activation of transcriptional programs by prospero homeobox-1 (Prox1) and the SRY-related HMG-box family member Sox18 [5]. Finally, circulating endothelial progenitor cells (EPCs), which can be experimentally isolated and differentiated into highly proliferating endothelial colony-forming cells (ECFCs), are thought to aid postnatal vasculogenesis and to contribute to the regeneration of damaged vasculature [7]. 

The heterogeneity of ECs reflects tissue-dependent differences in their functionality. Meeting these different demands requires a certain morphological variability, which is partially manifested by regional differences in intercellular junctions [3]. The endothelium of arteries and veins is continuous, while the endothelium of capillaries may also be fenestrated or discontinuous depending on the different underlying needs of the tissue [8]. In most capillaries of the brain, lungs, and skeletal muscle, a continuous sheet of ECs is held together by tight junctions and is anchored to the basal membrane [3]. Other tissues build on a fenestrated endothelium with pores of around 60–80 nm in diameter that are spanned by a diaphragm, which increase permeability and allow small molecules to diffuse [7]. This type of endothelium is found in organs involved in filtration or secretion, including exocrine and endocrine glands, gastric and intestinal mucosa, choroid plexus, kidney glomeruli, and a subpopulation of renal tubules [3,8]. In contrast to the intact basal membrane in fenestrated endothelium, the underlying basement membrane in discontinuous endothelium is poorly formed and exhibits larger fenestration (100–200 nm) among ECs, which are devoid of diaphragm [9]. Discontinuous endothelium is found in the sinusoids of the liver, spleen, and bone marrow [3]. Other morphologic differences are seen in the size and thickness of ECs. Aortic ECs are 1 µm thick compared to the cells of capillaries, which are 0.1 µm thick, and veinous ECs are 0.1–0.2 µm [9]. ECs can be remodeled in response to hemodynamic shear stress [8]. Changed flow patterns do not only result in region-specific phenotypic differences but also lead to changes on a molecular level by modifying gene expression [5]. The special role of the endothelium as an interface between underlying tissue and blood or lymphatic fluid makes it an important contributor to the circulating secretome. Therefore, it can have an impact on the plasticity and adaptation of ECs in different vascular beds as well as on circulating immune cells [4]. Taken together, ECs in different vascular beds reflect their respective physiological roles, which meet tissue-specific demands, by highly adopted and therefore heterogeneous phenotypes. The question that remains is if those heterogeneities of endothelial cells are transferred to their respective extracellular vesicles.

## 2. Biology of Extracellular Vesicles

Extracellular vesicles (EVs) have been found to influence a variety of pathological and physiological processes, such as inflammation, coagulation, or atherosclerosis, specifically in the context of vascular biology. However, their small subfractions (<200 nm) especially, together with possible differences between different vascular origins, remain poorly understood to this day. In general, EVs are defined as lipid membrane-enclosed vesicles with cellular origin that transport bioactive cargo including lipids, proteins, and nucleic acids. Not considering their cellular origin or cargo, they are commonly differentiated based on their size and release pathway into exosomes (30–150 nm), microvesicles (100 nm–1 µm), and apoptotic bodies (50 nm–5 µm) as the three main subcategories [10]. The small subset of EVs, most often termed exosomes, are vesicles that are actively secreted via an endosomal pathway. After the inward budding of the plasma membrane during endocytosis, membrane-bound proteins as well as extracellular components are internalized [11]. Subsequently, after scission from the membrane, the internally formed vesicles, now termed early endosomes, experience further regulated inward budding and cargo sorting during the maturation to late endosomes. The regulated inward budding and transport of cytoplasmic cargo leads to the formation of intraluminal vesicles (ILVs), which hallmarks the transition of late endosomes to multivesicular bodies (MVBs) [12]. These sorting and vesicle-formation processes are a topic of ongoing research that has revealed the high complexity and involvement of a variety of different proteins such as the endosomal sorting complex required for transport (ESCRT) or RAS-related protein RAB31. Mature MVBs are either fused with lysosomes for content degradation by hydrolytic enzymes through a ubiquitin- and clathrin-dependent manner or are trafficked to the cell membrane, where the subsequent fusion causes the release of the ILVs into the extracellular space in which they are then termed exosomes [12,13,14,15,16,17,18]. The commonly larger species of EVs, termed microvesicles (MV), originate, in contrast to exosomes, from the budding of the plasma membrane into the extracellular space and subsequent dissociation from the membrane. Specific adaptation of physical properties by changes of lipid and protein components thereby allows the remodeling of the cellular membrane, leading to separation and release of the MVs. Various molecules such as the ARF6 GTPase or vesicle-associated membrane protein 3 (VAMP3) have been shown to facilitate the active transport of cargo, such as proteins, enzymes, as well as nucleic acids, to the membrane prior to the budding process [19,20,21]. Although similar in size, apoptotic bodies show distinct differences to their exosomal and microvesicular counterparts. Apoptotic bodies are generally released through internal changes of the cytoskeleton and increasing hydrostatic pressure during cell death. These vesicles mainly function as a means of disposal of the dying cell, resulting in a highly similar proteomic profile to the cell lysate itself. The release of apoptotic bodies has been shown to influence various biological processes, such as the activation and modulation of the immune system by transferring antigens, increasing cell proliferation in stem cells as well as tumor niche formation [10,22,23,24,25] 

## 3. Molecular Signatures of Endothelial Cells and Endo-EVs

Identifying molecular profiles that could serve as endothelial EV (endoEV)-specific biomarkers relies on solid evidence of tissue-specific signatures of parental ECs. In addition to the pan EC markers vascular endothelial (VE)-cadherin (CD144), platelet endothelial cell adhesion molecule-1 (PECAM-1, CD31), and van Willebrand factor [26], recent studies revealed further constitutively expressed markers in circulating precursors and mature ECs of different vascular beds. ECFCs express homing-associated cell adhesion molecule (HCAM, CD44), mast/stem cell growth factor receptor (SCFR, c-kit, CD117), endoglin (ENG, CD105), 5′-nucleotidase (NT5, CD73), and stemness-associated transcription factors Klf4, Oct4 and c-Myc [27,28,29,30,31]. Blood vascular endothelial cells (BECs) express neural (N)-cadherin (CD325), pathologische Anatomie Leiden-endothelium (PAL-E) reactive antigen, urokinase, versican, collagen 8A1, ENG, and endothelial cell specific molecule-1 (ESM-1, endocan) [26,31,32,33,34]. More specifically, PAL-E is characteristic for ECs of blood capillaries and small veins [35,36]. Lymphatic endothelial cells (LECs) also express lymphatic vessel endothelial hyaluronan receptor-1 (LYVE-1), VEGFR-3, neuropilin-2 (NRP-2), podoplanin (PDPN), Prox-1, forkhead box protein C2 (FOXC2), and integrin α9 [32,33,37,38,39]. Generally, ECs can differ in their marker expression in a tissue-specific manner or share certain markers across vascular beds. Highlighting just a few uniquely expressed BEC markers, glucose transporter type 1 (GLUT-1) and integral membrane protein 2A (ITM2A) are seen to be specific to brain vascular ECs in mice, just as fatty acid binding protein 4 (FABP4) is to coronary vessel ECs and natriuretic peptide receptor C (NPR3) and cytokine-like protein 1 (C17) are to endocardial ECs [40]. VEGFR-3 is widely accepted to be LEC-specific although also expressed in BECs, constituting fenestrated capillaries in bone marrow, liver, spleen, and kidney glomeruli [37]. LECs that line different lymph node regions differentially express LYVE-1 [41]. The intricacy of EC marker expression profiles reflects the need for combinatorial detection strategies. This is equally important for characterizing endoEVs from different vascular beds in different host tissues and under varying stimuli.

**Figure 1:**
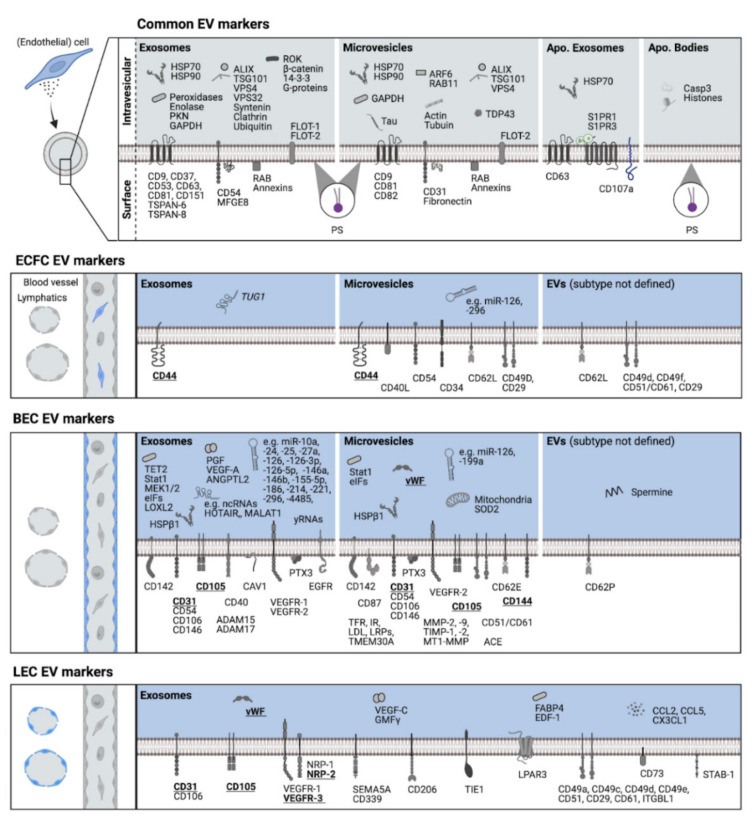
Overview on molecules commonly expressed with EVs of different biogenesis pathways and molecules found in association with EVs from endothelial colony-forming (progenitor) cells (ECFCs) and ECs of blood (BEC) and lymphatic vascular beds (LEC). Underlined bold molecules indicate parental cell markers. Common EV markers [5,42,46,77]; ECFC EV markers [29,48,49,50,51]; BEC EV markers [44,52,53,54,55,56,57,58,61,62,63,64,66,67,68,69,70,71,72,73,75,78,79,80,81,82]; and LEC EV markers [76]. ACE, angiotensin converting enzyme; ADAM15/17, disintegrin and metalloproteinase domain-containing protein 15/17; ALIX, apoptosis-linked gene (ALG)-2 interacting protein X; ANGPTL2, angiopoieitin-like protein 2; ARF6, ADP-ribosylation factor 6; Casp3; caspase 3; CAV1, caveolin 1; CCL2/5, chemokine ligand 2/5; CD, cluster of differentiation; CX3CL1, fractalkine; EDF-1, endothelial differentiation-related factor-1; EGFR, epidermal growth factor receptor; eIFs, eukaryotic initiation factors; FABP4, fatty acid binding protein 4; FLOT-1/2, flotillin 1/2; GAPDH, glyceraldehyde-3-phosphate dehydrogenase; GMFγ, glia maturation factor gamma; HSP70/90, heat-shock protein 70/90; HSPß1, heat-shock protein ß1; IR, insulin receptor; ITGBL1, integrin subunit beta-like 1; LDL, low-density lipoprotein; (l)ncRNA, (long) non-coding RNA; LOXL2, lysyl oxidase-like 2; LPAR3, lysophosphatidic acid receptor 3; LRPs, low-density lipoprotein receptor-related proteins; MEK1/2, mitogen-activated protein kinase 1/2; MFGE8, milk fat globule-epidermal growth factor 8 protein (lactadherin); miR, micro RNA; MMP-2/-9, matrix metalloproteinase-2/-9; MT1-MMP, membrane-type 1 matrix metalloproteinase; NRP-1/-2, neuropilin-1/-2; PGF, placental growth factor; PKN, protein kinases; PS, phosphatidylserine; PTX3, pentraxin-related protein; RAB/RAB11, Ras superfamily of small G proteins (GTPases); ROK, Roh-associated kinases; S1PR1/3, sphingosine-1-phosphate receptor 1/3; SEMA5A, semaphoring 5A; SOD2, superoxide dismutase 2; STAB-1, stabilin-1; Stat1, signal transducer and activator of transcription-1; TDP43, transactive response DNA binding protein 43; TET2, Tet methylcytosine dioxygenase 2; TFR, transferrin receptor; TIE1, tyrosine kinase with immunoglobulin-like and EGF-like domains 1; TIMP-1/-2, tissue inhibitor of matrix metalloproteinase-1/-2; TMEM30A, transmembrane protein 30A; TSG101, tumor susceptibility gene 101 protein; TSPAN-6/8, tetraspanin 6/8; TUG1, taurine upregulated gene 1 long non-coding RNA (lncRNA); VEGF-A/-C, vascular endothelial growth factor-A/-C; VEGFR-1/2/3, vascular endothelial growth factor receptor-1/-2/-3; VPS4/32, vacuolar protein sorting-associated protein 4/32; vWF, van Willebrand factor; yRNA, small non-coding RNA; 14-3-3, signaling protein superfamily.

EndoEVs as liquid biopsy-derived biomarkers are increasingly coming into focus as their abundance and cargo provide valuable information on EC response to certain stimuli and can be pathognomonic for EC damage. EndoEVs can be characterized by markers inherent to their biogenesis or parental cells or those accumulating under certain conditions (Figure 1). Contrary to common assumptions, phosphatidylserine (PS) is not only found in the outer membrane leaflet of MVs but also exosomes [42]. PS on endoEVs facilitates tethering to distant ECs and conveys pro-coagulant effects, as was shown by tissue factor (CD142)-bearing BEC-EVs [43,44]. Recent evidence suggests an apoptosis-related subtype of exosomes (apoExos) that in BECs are shed in a caspase 3-dependent manner [45]. ApoExos, alongside exosome-specific tetraspanin CD63, express the lysosomal marker LAMP1 (CD107a), heat-shock protein 70 (HSP70), and sphingosine 1-phosphate receptors 1 and 3 (S1PR1, S1PR3) [46,47]. BEC apoExos also enrich pro-inflammatory non-ribosomal non-coding viral-like RNAs [45].

ECFC-EVs constitutively express HCAM (CD44), which is intrinsic to their parental cells [29,48], and further reveal an expression of certain adhesion molecules, e.g., hematopoietic progenitor cell antigen (CD34), intercellular adhesion molecule-1 (ICAM-1, CD54), L-selectin (CD62L), integrins α4 (CD49d), α6 (CD49f), ß1 (CD29), and αvß3 (CD51/61), but also CD40 ligand, which is involved in activation of antigen presenting cells [48,49,50]. ECFC-exosomes exert anti-inflammatory effects through lncRNA TUG1-mediated alteration of macrophage histone deacetylation [51]. ECFC-MVs convey pro-angiogenic signals via transfer of miR-126 and miR-296, while specific sorting of further miRNAs to MVs was also described [49].

BEC-EVs were found to express EC markers VE-cadherin, PECAM-1, vWF, and ENG [52,53,54,55,56,57,58,59,60,61]. In addition, angiogenesis-implicated growth factor receptors VEGFR-1 and VEGFR-2 and their ligand VEGF-A were detected, with the latter’s expression being increased under hypoxic conditions [52,62,63]. VE-cadherin expression on BEC-EVs is also primed under hypoxia [55,57]. PECAM-1 and ENG were upregulated on BEC-EVs upon growth factor depletion-mediated EC apoptosis [54]. While ENG is downregulated in blood—brain barrier ECs under TNF stimulation, it is highly upregulated in their MVs but consistently expressed in exosomes [56]. TNF pro-inflammatory stimuli further exceeded baseline expression of the cell adhesion molecules intercellular adhesion molecule-1 (ICAM-1, CD54), vascular cell adhesion molecule-1 (VCAM-1, CD106), and E-selectin (CD62E), the classical complement pathway activator pentraxin-related protein (PTX3), and the transcription factor signal transducer and activator of transcription 1 (STAT1) in both BEC-MVs and -exosomes [54,55,56]. MVs from blood—brain barrier ECs also increased expression of eukaryotic translation initiating factors (eIFs) and cell-death-preventing superoxide dismutase 2 (SOD2) [56]. As a transcription regulator, Tet methylcytosine dioxygenase 2 (TET2) was found in BEC-exosomes and is downregulated by CD137 TNF receptor signaling [64]. EV-mediated transfer of CD142 by TNF-stimulated BECs was linked to marked coagulation factor activation [44] although BECs line up an otherwise hemocompatible interface between blood and extravascular tissue. As widely described for CD142 in MSC-derived therapeutics [65], BEC-EVs may pose a similar procoagulant risk when administered intravascularly, necessitating proper endoEV characterization. MVs from brain-BECs express receptors that transfer molecules across the blood—brain barrier, i.e., transferrin receptor, insulin receptor (CD220), low-density lipoproteins (LDL), LDL receptor-related proteins (LRPs), and transmembrane protein 30A (TMEM30A) [66]. Further adhesion molecules found on BEC-EVs are melanoma cell adhesion molecule (MCAM, CD146), P-selectin (CD62P), and integrin αvß3 [53,54,55,58]. BEC-EVs were also shown carrying matrix degrading metalloproteinases, i.e., ADAM15 and ADAM17 in exosomes [56] and MMP-2, MMP-9, and MT1-MMP in MVs [67]. Furthermore, metalloproteinase inhibitors TIMP-1 and TIMP-2 could be found [67]. EVs from hypoxic BECs were also shown to promote extracellular matrix crosslinking via lysyl oxidase-like 2 (LOXL2) transfer [68]. BEC-exosomes further express pro-angiogenic molecules, e.g., angiopoietin-like protein 2 (ANGPTL2) [69]. Pro-angiogenic effects of BEC-EVs could also be observed due to transfer of miR-24, miR-214, and miR-126 [61,70,71,72]. Beside cardioprotective miR-126 and miR-199a [61] and pro-proliferative miR-25, miR-186, and miR-221 [71], BEC-EVs transfer a plethora of further miRNAs, yRNA fragments [71], and lncRNAs [73]. Cardioprotective EV properties could be hampered by caveolin-1 exosomes from interleukin-3 (IL-3)-stimulated BECs [74]. In vitro, EC morphology also regulates inflammatory response, as EVs from elongated BECs carry markedly higher amounts of anti-inflammatory miR-10a [75]. Over and above this excerpt of molecules, RNA sequencing and proteomic studies on BEC-EVs revealed a variety of further RNAs and proteins, respectively [52,56,71]. 

In LEC-EVs, there is sparse information on molecular patterns. In one proteomic study using exosomes from primary human LECs, more than 1700 proteins were detected [76]. Beside vWF, PECAM-1, and ENG, in this study, LEC markers VEGFR-3 and NRP-2 (also NRP-1) and the lymphangiogenic factor VEGF-C were also found. Further adhesion molecules, e.g., VCAM-1 and integrins α1, α3, α4, α5, αV, ß1, ß3, and subunit beta-like 1, could also be detected. Treatment of LECs with TNF resulted in an overall motility-promoting protein signature [76]. In conclusion, further studies are needed to elucidate additional and specific molecular signatures for different vascular bed-derived endoEVs.

## 4. The Role of EndoEVs in Pathology

The secretion of EVs represents one form of cell—cell communication and is thus an important component in physiological and pathological processes [5,83]. Although little is known about the exact identity of endoEVs, recent studies suggest a high degree of heterogeneity and plasticity influencing cells, tissues, and organs both locally and systemically [5,84]. EndoEVs exhibit molecular patterns, which suggests that they contribute to the maintenance of tissue homeostasis, including cell survival and protection, angiogenesis, and an anti-inflammatory state. Moreover, through the production of nitric oxide (NO) ECs are able to control vasodilation, vasoconstriction, and thrombogenesis. In this context, it has been shown that the endothelial NO release triggered by high shear stress can impair the release of endoEVs [83]. A recent study investigated the effect of impaired blood flow on endothelial MV release in healthy subjects using an occlusion cuff model. In comparison to the control arm, significantly higher levels of CD62E+ and CD31+/CD42b- endoMVs were observed [85]. Accordingly, the levels of circulating endoEVs in the physiological state are considered to be rather low [83,86]. Nonetheless, EndoEVs exhibit molecular patterns that suggest that they contribute to the maintenance of tissue homeostasis, including cell survival and protection, angiogenesis, and an anti-inflammatory state. Other studies provide evidence that exosomes derived from ECs exposed to hypoxia and inflammatory cues contain proteins and mRNA that indicate the state of the cell of origin [87]. In addition, ECs have been shown to actively protect themselves from apoptosis and cellular stress by releasing endoMVs. This is achieved by the encapsulation of intercellular caspase-3 into endoMVs and their subsequent release from ECs, which results in a reduction of respective molecule levels within the cell [88]. Moreover, by presenting the endothelial protein C receptor, endoMVs can accelerate the activation of protein C, which may have anti-inflammatory and anti-apoptotic effects and thereby promote cell survival [89]. By transferring miRNA-rich microvesicles, protein and mRNA expression in recipient cells has been shown to be influenced by endoEVs [90]. For example, the horizontal transfer of miR-126 can inhibit the expression of SPRED-1 (an intracellular inhibitor of angiogenic signaling), leading to the promotion of angiogenesis [91]. Additionally, endoMVs were shown to express the urokinase-type plasminogen activator (uPA) and its respective receptor (uPAR). Therefore, endoMVs may contribute to the generation of plasmin, which in turn can have beneficial effects on tissue remodeling and in vitro tube formation [78]. Matrix metalloproteinase-2 and -9 in secreted endothelial vesicles can be associated with degradation of the surrounding extracellular matrix, release of growth factors, and thereby promotion of angiogenesis [67]. Some studies indicate that these effects are dose-dependent, with low (physiological) concentrations of endoMVs appearing to favor the formation of capillary-like structures and high concentrations inhibiting tube formation [78]. EndoEV-dependent transfer of functional miRNA-222 into ECs can decrease the expression of ICAM-1, which appears to have anti-inflammatory effects [92]. Moreover, endoEVs containing anti-inflammatory miRNAs are able to limit monocyte activation [93]. In contrast to the observation that rather low levels of endoEVs can be detected in physiological conditions, elevated levels of endoEVs may be indicative of diseases associated with endothelial dysfunctions [83]. Increased amounts of endoEVs are produced and released upon activation or apoptosis of endothelial cells. These EVs may play a role in the onset and progression of various vascular diseases [83]. Activating stimuli for ECs include proinflammatory cytokines such as TNF-a, bacterial lipopolysaccharide, reactive oxygen species (ROS), plasminogen activator inhibitor, thrombin, C-reactive protein, low shear stress [94], hypoxia, cell injury, and senescence [95]. EndoEVs released in response to proinflammatory signals may further drive inflammation through paracrine signal transduction, thereby promoting endothelial dysfunction [5]. These findings were further demonstrated by showing that endoMVs were able to activate human pulmonary microvascular endothelial cells and to induce the production of proinflammatory cytokines [96]. EndoEVs released upon TNF-α stimulation showed a significant change in the amount of proinflammatory mRNA, such as IL-8, MCP-1, IL-32, and VCAM-1, the latter being involved in the mobilization of leukocytes, which in turn also favors a proinflammatory state [83,97]. Endothelial dysfunction is a critical feature of type 2 diabetes and is considered a major cause of diabetic cardiovascular complications [98]. Koga et al. observed that significantly elevated levels of CD144^+^endoMVs were found in patients suffering from diabetes mellitus compared to nondiabetic controls. In addition, they described that the CD144^+^endoMVs levels in diabetes mellitus patients with coronary artery disease (CAD) were significantly higher than in diabetic patients without CAD [98].

In addition, endothelial dysfunction can be associated with the development of atherosclerosis [90]. EndoEVs carrying miR-155 were able to enhance the activation of monocytes and shift the balance from an anti-inflammatory to a pro-inflammatory phenotype, causing these EVs to contribute to atherosclerosis [99]. Partially, endoEVs released by inflammatory stimuli can be characterized by co-expression of tissue factor (TF) and phosphatidylserine (PS) on their outer surface, which facilitates the binding of EVs and provides them with procoagulant properties. Thus, the release of endoEVs seems to be associated with the activation of coagulation cascades and the formation of thrombi, suggesting that they might be also be linked to the development of stroke [86,90]. Simak et al. showed higher PS^+^endoMVs levels in patients with acute ischemic stroke compared to controls. A correlation between endo-EV level and lesion volume and clinical outcome was observed [100]. Additionally, it was found that elevated plasma levels of endoMVs can be linked to acute coronary syndrome, which includes myocardial infarction, angina pectoris, and myocardial ischemia. [55].

## 5. Conclusions

Taken together, increasing evidence supports the potential roles of EVs derived from blood and lymphatic endothelial cells in order to maintain physiological homeostasis as well as their roles in pathological settings. Further studies specifically addressing the vascular bed-specific molecular cargos in endoEVs will be needed to clarify their various roles and functions throughout biological processes. To achieve this goal, special attention must be given to the isolation and characterization of EVs, which demands general standards in EV research. As importantly corroborated by the International Society of Extracellular Vesicles (ISEV), publishing guidelines to help researchers in this fast-growing field studies on crucial issues, such as the comparability of EV, are of importance beyond any specificity of EVs in the vascular system [101].

## Data Availability

Not applicable.
